# Sustainability in Jordan’s Municipal Solid Waste Management System: Reduction of Methane Emissions and Benefits to Public Health

**DOI:** 10.21203/rs.3.rs-9283805/v1

**Published:** 2026-04-13

**Authors:** Anas Ghawanmeh, Fayez Abdulla, Marc G. Weisskopf, Wael K. Al-Delaimy, Mark R. Jacobsen, Sarah Al-Omoush, Alham Al-Shurafat

**Affiliations:** 1 Department of Civil Engineering, Jordan University of Science and Technology (JUST), Jordan,; 2 Department of Environmental Health, Harvard T.H. Chan School of Public Health, Boston, Massachusetts, USA,; 3 Herbert Wertheim School of Public Health and Human Longevity Science, University of California,San Diego, San Diego, California, United States of America.; 4 Department of Economics, University of California, San Diego, San Diego, CA, USA.; 5 Department of Environmental Engineering, North Carolina State University (NCSU), Raleigh, NC, USA,

**Keywords:** Municipal Solid Waste Management, Methane, LEAP, WasteMap, Jordan, scenarios, SLCPs

## Abstract

Municipal solid waste management (MSWM) in Jordan faces significant challenges. Jordan’s primary waste management strategy relies heavily on final disposal, with up to 80% of all generated solid waste ultimately being landfilled. These unsustainable practices lead to environmental degradation and public health risks. Increased methane emissions have harmed air quality: methane can lead to the formation of ground-level ozone, which in turn causes respiratory problems including asthma and pneumonia. The main objective of this study is to evaluate the impact of improved solid waste management strategies on methane emissions in the period from 2020 to 2050. The Low Emissions Analysis Platform (LEAP) is used for the analysis, and current methane emissions from municipal solid waste are estimated using data obtained from the Ministry of Local Administration, the Greater Amman Municipality, the Department of Statistics, and the World Health Organization. These datasets, which include information on waste quantities and landfill boundaries, are integrated into the LEAP software to facilitate estimation of methane emissions. Our analysis finds that, without intervention, methane emissions will rise from 4.3 million metric tons of carbon dioxide equivalent in 2020 to 7.8 million tons by 2050 due to increasing population and economic expansion. In addition to the serious environmental consequences, we estimate public health damages of $41 billion per year by 2050, linked to excess mortality. In the short-term, methane emissions can be mitigated by following a “circular economy” strategy of reuse and recycling. This could reduce methane emissions by 12% by 2030. Medium-term strategies, including the establishment of sanitary landfills and recycling facilities, combined with the short-term projects can reduce methane emissions 34% below baseline by 2040. Adding long-term strategies, including composting and additional reuse, leads to reductions of 55% by 2050. More dramatic individual changes to the system also have large effects: shifting to incineration instead of landfills reduces methane emissions by 52%. Advanced recycling facilities can reduce emissions by 35%. These results highlight the impact of improved integrated waste management approaches on reducing emissions and thus enhancing public health and global climate goals. The paper underscores the importance of technical advancement in solid waste management in Jordan. We show that large environmental and economic gains are possible with aggressive governmental actions and collaboration among stakeholders.

## 1 Introduction

Municipal solid waste management (MSWM) is a critical issue, especially in low- and middle-income countries (Kuylenstierna et al., 2020; Chiemchaisri & Visvanathan, 2008). In Jordan, MSWM is a pressing concern (Gautam & Agrawal, 2020). Jordan faces serious waste management issues as a result of economic growth, growing population, and the expansion of the industrial and agricultural sector (JGBC, 2016). Jordan produces more than 3 million tons of municipal solid waste annually and this amount is expected to increase by 3% annually (GIZ, 2014). It is estimated that more than 90% of waste ends in waste landfills (Gibellini et al., 2022), causing a wide range of environmental challenges, including carbon dioxide and methane emissions, as well as other air, soil, and water pollution (UNEP and WMO, 2011). Globally, 5% of total greenhouse gas emissions are from solid waste (UNEP, 2017; Bogner et al., 2008). In addition to its high global warming potential (methane’s impact is 28 times more significant than that of carbon dioxide over 100 years (Change, 2023)) methane from landfills creates important public health harms (WHO, 2014).

The current waste management system in Jordan is unable to handle the growing amounts of waste. Just two of the country’s eighteen known landfills are sanitary, the largest is Al-Ghabowi which receives waste from the capital and neighboring cities. Jordan also has a low recycling rate of 5–10% due to a lack of infrastructure for waste sorting; most recycling happens in the private or informal sectors (Saidan et al., 2016).

Jordan’s environment and public health are at risk due to improper solid waste management both during collection and disposal. The waste management system has been under added strain as immigrant populations rise, now making up about 30.6% of the population (DOS 2024, Al-Shurafat et al., 2025; Pohlmann, 2017). The Jordanian government allocates about 90 million dinars annually to waste management with municipalities, and 70 % of this is in cost recovery causing a financial burden (Nassour et al. 2008). The problem is made worse by the absence of incentives and a financial mechanism, which prevents the adoption of sustainable waste management practices (Ikhlayel et al.,2016).

Sustainable waste management is essential to creating thriving cities and communities according to the Sustainable Development Goals (SDGs), especially SDG 11, which focuses on cities and communities (Abu Hajar et al., 2020; Mittal et al., 2017; Premakumara et al., 2018). Proper MSWM is critical to public health, reducing air pollution, and mitigating climate change (Lee et al., 2023; WHO, 2018).

Our aim in this study is to estimate the quantity of short-lived climate pollutants (SLCPs), particularly methane, associated with different solid waste activities in Jordan and to measure them according to international best practices and sustainability standards. We use the Low Emissions Analysis Platform Integrated Benefits Calculator (LEAP-IBC) and the Waste Methane Assessment Platform (WasteMAP) tracking tool to estimate the methane generation from landfills and the reductions achievable with alternate technologies (Shindell et al., 2012).

By using a comprehensive approach that combines modeling techniques with emission tracking this study aims to identify the best strategies for reducing methane emissions from municipal solid waste management in Jordan. These initiatives will improve public health outcomes and also aid global efforts to mitigate climate change (Kumar & Sharma, 2013). This study also provides estimates of the public health benefits available through reductions in methane emissions.

## 2 Materials and Methods

This study looks at Jordan’s MSWM system with a focus towards mostly reducing methane (CH_4_) emissions and assessing their prospective public health effects. Starting with data collection and baseline assessment, the approach is methodically explored through emissions inventory and modelling, scenario development, health and environmental impact assessment, scenario comparison, policy recommendations, and stakeholder involvement. Every phase provides a complete framework for assessing current waste management practices, estimating future trends, and identifying environmentally appropriate mitigating solutions.

With an extensive database of technology and environment, The Low Emissions Analysis Platform (LEAP) can provide competent information of describing the technical characteristics and environmental impacts of energy technologies and estimating emissions of greenhouse gases and short-lived air pollution from all their sources in key sectors, (Heaps, 2020). LEAP serves as the foundation for estimating emissions of pertinent greenhouse gases (GHGs), short-lived climate pollutants (SLCPs), and air pollutants from all principal source sectors. The Low Emissions Analysis Platform - Integrated Benefits Calculator (LEAP-IBC) provides a model framework for estimating the public health and climate change impacts of pollutants and is trusted by most international bodies and used to guide policymakers through assessing the impacts of proposed decisions (Kuylenstierna et al., 2020).

**fig. 1:**
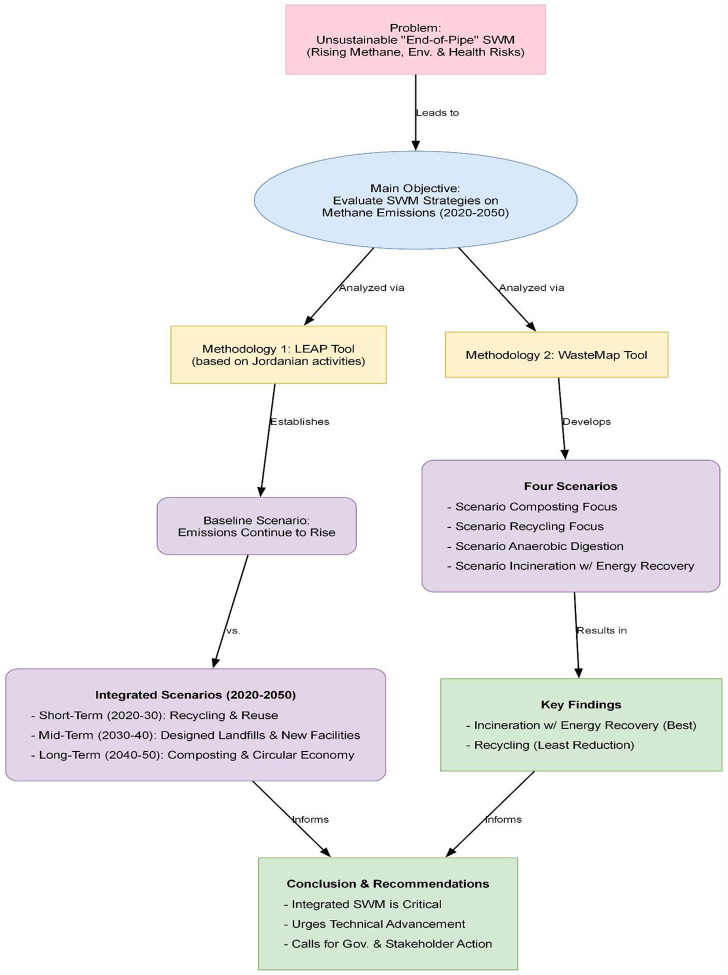
Research Roadmap: Sustainable Waste Management Jordan

These estimates encompass an emission inventory for historical years, together with prospective scenarios regarding the potential evolution of emissions under various alternative conditions and Figure 1 shows the methodology of this study.

### 2.1. Data Collection

In the first phase—data collecting and baseline assessment—reliable statistics on municipal solid waste generation, management practices, and related emissions were gathered from key governmental and international sources including the Ministry of Local Administration (MoLA), the Greater Amman Municipality (GAM), the Department of Statistics (DOS), and the World Health Organization (WHO). Covering 2019 through 2023, the dataset comprises waste quantities and disposal methods (Table 1). The year 2020was chosen as a baseline for this study, due to the availability of all the information required for the study, and accurate data collection by the Ministry of Environment from the concerned authorities in Jordan.

Complementing these numbers—as shown in Table 2 are demographic, economic, and health data to provide a full picture of Jordan’s socioeconomic and public health environment for waste management. Supporting data helps one forecast the increase in SLCP emissions and assess their effects. Tables 2 below provide economic, demographic, and projected Respiratory Disease Ozone Relative Risk Rate 1.04 for Jordan based on The Institute for Health Metrics and Evaluation (IHME) supplied Jordan’s age-specific death statistics for 2019 and population age structures (Cao et al., 2019).

### 2.2. Emissions Inventory

In the second phase, an emissions inventory was created using the Low Emissions Analysis Platform (LEAP) to estimate methane emissions resulting from landfill activity and waste treatment operations. The Low Emissions Analysis Platform (LEAP) projected emissions under several conditions, including population growth and expected advancements in waste management technology. Key elements, including waste volume, the fraction of degradable organic carbon, degradation rates, methane emission factors, and methane recovery rates, were included in the Total Annual Methane Emissions (TAME) equation to project methane emissions (US EPA, 2025). The models output calculated the potential for mitigation (e. g. the projected total annual methane emissions under each scenario). showing that emissions could be reduced by about 30% under the advanced recovery scenario. and determined the factors that influence sensitivity (e. g. A. content of organic waste) creating a standard by which to measure the effectiveness of intervention tactics.

We estimated methane emissions from (MSWM) by means of LEAP-Model (Low Emissions Analysis Platform) by computing the factors, including waste diversion rates, recycling efforts, and other elements. The model takes into account important parameters such as the amount of MSW landfilled or dumped at site *i* each year (MSWi measured in Gg), the percentage of waste that is degradable organic carbon (DOC), and the percentage of DOC that degrades the methane emission factor and the methane-to-carbon conversion ratio. To improve our estimates, we also take into consideration the yearly methane recovery at landfill locations. In Jordan’s MSW management framework we apply various waste management scenarios to evaluate their impact on sustainability and public health by determining their potential to lower methane emissions. To identify emissions of short-lived climate pollutants (SLCPs) such as methane linked with various solid waste activities. The data gathered corresponds to the volume of waste buried in a landfill. Equation 1 allows us to predict the amount of methane emissions resulting from solid waste disposal (Pipatti et al., 2006).

$$\text{TAME}=\sum_{i}\left[MSW_{i}\cdot FDOC_{i}\cdot FD_{i}\cdot MEF_{i}\cdot CR_{i}-MR_{i}\right]$$

Where:

- **MSW**_**i**_: the annual amount of MSW landfilled/dumped at site i (in Gg).
- **FDOC**_**i**_: the fraction of degradable organic carbon (DOC) in the MSW.
- **FD**_**i**_ : the fraction of DOC that degrades.
- **MEF**_**i**_: the methane emission factor.
- **CR**_**i**_: the methane/carbon conversion ratio.
- **MR**_**i**_: the annual methane recovered at site i (in Gg).

To deliver a thorough study of emission reduction potential, the scenarios were divided into three temporal periods, illustrating the expected progression of waste management methods in Jordan from 2020 to 2050 as shown in table 3. The stages were simulated using LEAP to forecast emissions and evaluate the effects of mitigation methods throughout time.

A key component of the study’s methodology was determining baseline emissions, which serve as a benchmark for analyzing mitigation tactics. Measuring methane emissions from important solid waste management processes such as waste treatment incineration and sanitary landfilling was part of the methodology. Emissions under a business-as-usual scenario were projected using the LEAP model, which depicted current practices and their anticipated changes through 2050. Improved waste management techniques could potentially reduce emissions (Napia et al., 2012), and three different mitigation scenarios were created to investigate this possibility. The approach was broken down into three time-frame scenarios that matched the projects that were anticipated to be carried out in Jordan. The first scenario which examines early projects and potential enhancements spans the years 2020–2030. The second scenario focuses on medium-term projects and evaluates their impact spanning the years 2030–2040. The time frame for the third scenario is 2040–2050 and evaluates long-term projects and their effects on emissions of methane.

Five distinct waste management scenarios were developed to explore Jordan’s potential for reducing methane emissions by using the Waste MAP platform.

WasteMAP is used to estimate and reduce methane emissions from landfills. Its scenario-analysis tools were used to model five waste management strategies for Jordan, assessing the potential for methane reduction through practices such as waste diversion composting and landfill gas capture. The projections for methane emissions in Jordan’s waste management scenarios were modelled utilizing the first-order decay (FOD) approach as outlined in the IPCC Guidelines for National Grenhouse Gas Inventories (IPCC, 2006). Methane emissions (CH_4_) from landfills were computed as follows equation (2):

$$\text{C}{\text{H}}_{4}=\sum_{x}\left[\text{MS}{\text{W}}_{\text{x}}\cdot \text{MCF}\cdot \text{DOC}\cdot \text{DO}{\text{C}}_{\text{f}}\cdot \text{F}\cdot \frac{16}{12}\cdot (1-\text{R})\cdot (1-\text{OX})\right]$$

Where :

**CH4**: Methane Emissions (The annual mass of CH4 emitted to the atmosphere)

**MSWx**: Mass of Municipal Solid Waste deposited in year x

**MCF**: Methane Correction Factor (Adjusts for site management; ranges from 0.4 for unmanaged shallow to 1.0 for managed anaerobic sites)

**DOC**: Degradable Organic Carbon (The fraction of MSWx that is carbon and can potentially decompose)

**DOCf**: Fraction of DOC that decomposes (The portion of DOC that ultimately decomposes under anaerobic conditions)

**F**: Fraction of CH4 in the landfill gas (The volumetric fraction of methane in the total landfill gas, typically 0.5)

**R**:Methane Recovery Factor (The fraction of generated CH4 that is captured and recovered or flared)

**OX**: Methane Oxidation Factor (The fraction of CH4 oxidized by microbes as it passes through the landfill cover before emission)

Scenario-specific diversion rates (e.g., organic waste to composting, non-recyclables to incineration) diminished municipal solid waste inputs to landfills, consistent with empirical research (IPCC, 2006). Energy recovery from incineration and anaerobic digestion adhered to mass-balance models (Yang, He and Fu, 2014b), whereas composting efficiency was derived from previous literature (Kumar, 2010).

These scenarios were created to depict a range of waste diversion and treatment techniques, including landfill gas capture, anaerobic digestion, composting, recycling, and energy recovery incineration. Each scenario was defined by distinct parameters, including waste diversion rates, adoption of treatment technologies, and effectiveness of landfill gas capture. The scenarios were created and utilized by WasteMAP, a tool intended for waste management planning and emissions monitoring.

### 2.3. Health Implications

There are a range of methods available for measuring the burden of disease in economic terms: LEAP leverages the commonly used approach of assigning a value of a statistical life (VSL) and focusing on the mortality impacts of pollution, primarily methane in this case. The VSL itself is constructed by assessing the trade-offs people are willing to make to reduce their risk of death (Viscusi and Masterman, 2017).

Importantly, the VSL is therefore not a value assigned to any specific lives, but rather a value placed on small reductions in relative risk which, when applied over a large population, lead to changes in overall mortality. Since the VSL is obtained by observing a person’s actual willingness to pay (WTP) for a small reduction in their mortality risk it is necessarily linked to income. Robinson et al. (2019) show in their Eq. 3 how to adjust the VSL for any target income level:

$$VSL_{\text{target}}=VSL_{\text{reference}}\times {\left(\frac{\text{Incom}e_{\text{target}}}{\text{Incom}e_{\text{reference}}}\right)}^{\text{elasticity}}$$

Where **VSLtarget** is the appropriate VSL to use for the target level of income and **VSLreference** is a reference VSL derived from an original empirical study. These are often performed in a high-income country or come from a meta-analysis. The VSLs are all reported in currency units. **Income**_**target**_ is the income in the target economy, in this case Jordan. **Income**_**reference**_ is income in the population studied to produce the reference VSL. **Elasticity** is the key parameter in the adjustment process and controls the rate at which VSL rises with income.

## 3. Results

### 3.1 Baseline Waste Management and Methane Emissions in Jordan.

Significant variations in the amounts of waste disposed of are revealed by Jordan waste landfilling trends between 2019 and 2023. The decline to 3.189 million tons in 2023 after the peak of 4.37 million tons of landfilled waste annually in 2022 points to variability influenced by policy, demographic, or economic factors. Jordan’s population is forecasted to reach 20.8 million and GDP projected at 96.8 billion USD by 2050.

**Fig 2:**
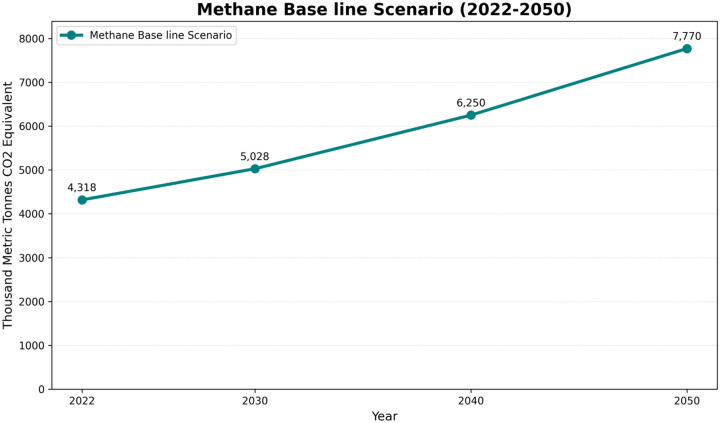
100-Year GWP: Direct (At Point of Emissions) Scenario: baseline scenario, All Fuels, Effect: Methane

Based on the LEAP platform and expected economic advancement and population increase, methane emissions from municipal solid waste landfills are anticipated to rise markedly, from 4.32 million metric tons of CO_2_ equivalent in 2020 to 7.77 million metric tons by 2050 as shown down in table 4 and figure 2 inventory of Methane Baseline Scenario.

### 3.2 Time-Framed Scenarios Using LEAP

We used the LEAP (Long-range Energy Alternatives Planning) model to study projected methane emissions from municipal solid waste (MSW) in landfills under three mitigation scenarios and a Baseline Scenario (BLS) approach. The Baseline scenario reflected the status of waste management as well as its anticipated development by 2050. Three time periods of mitigation scenarios were chosen: short-term (2020–2030), mid-term (2030–2040), and long-term (2040–2050), through ongoing and prospective projects, in consultation with the relevant ministries about these initiatives. For each scenario the potential impact of specific waste management programs on methane emissions measured in a thousand metric tons of CO_2_ equivalent was evaluated. As shown in table 5.

#### 3.2.1 Short-Term Scenario (2020–2030)

**Fig 3:**
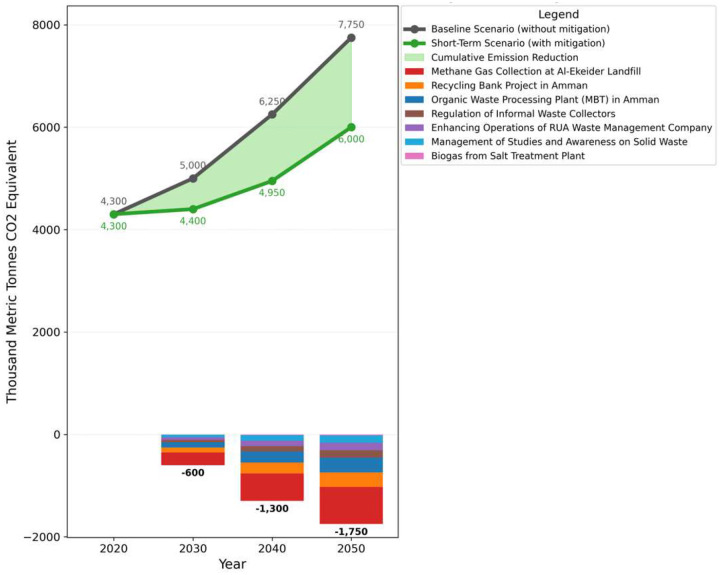
100-Year GWP: Direct (At Point of Emissions) Scenario: short term scenario, All Fuels, Effect: Methane

The baseline emissions for 2022 were 4,317.58 thousand metric tons of CO_2_ equivalent, anticipated to rise to 5,028.02 by 2030. Important projects for methane reduction includes the Organic Waste Processing Plant (MBT) and the Recycling Banks Project in Amman which will reduce emissions by 100 thousand metric tons of CO_2_ annually starting in 2030 as shown in figure 3. An annual reduction of 52 thousand metric tons was achieved through the regulation of informal waste collectors and the improvement of RUA waste management operations. The Methane Gas Collection at Al-Akeidar Landfill significantly reduced emissions by 251.40 thousand metric tons per year. Additionally, a yearly decrease of 52 thousand metric tons was made possible by the Management of Studies and Awareness of Solid Waste. Together these projects reduced emissions from 5028.02 to 4424.65 thousand metric tons by 2030 indicating a significant improvement in the short-term reduction of methane emissions.

#### 3.2.2 Mid-Term Scenario (2030–2040)

**Fig 4:**
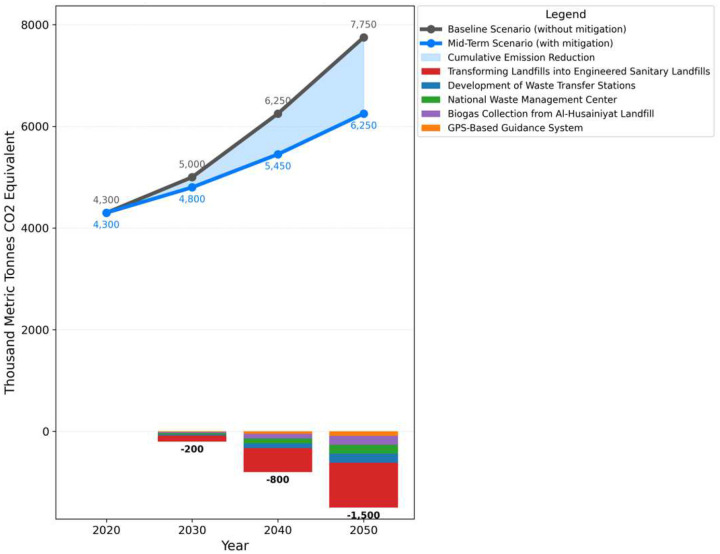
100-Year GWP: Direct (At Point of Emissions) Scenario: Mid-term scenario, All Fuels, Effect: Methane

The mid-term scenario focused on more advanced waste management techniques, building on the short-term projects. It is expected that emissions will have increased from the baseline of 5028.02 thousand metric tons in 2030 to 6250.37 thousand metric tons in 2040. Turning landfills into Engineered Sanitary Landfills was one of the major initiatives lowering emissions by 251.4 thousand metric tons in 2030 and lowering it to 312.52 by 2040. In 2030, the construction of waste transfer stations helped reduce 50.28 thousand metric tons. By 2040, that number had risen to 62.50. Emissions were decreased by 25.14 thousand metric tons in 2030 and 31.25 thousand metric tons by 2040 thanks to the GPS-Based Guidance System. The National Waste Management Center and Al-Husainiyat Landfills Biogas Collection both made contributions to the 2030 reduction of 50.28 thousand metric tons and 62.50 by 2040. The cumulative impact of these projects showed that mid-term waste management strategies were effective as emissions decreased from the baseline of 6250.37 to 5719.09 thousand metric tons by 2040 as shown in figure 4.

#### 3.2.3 Long-Term Scenario (2040–2050)

**Fig 5:**
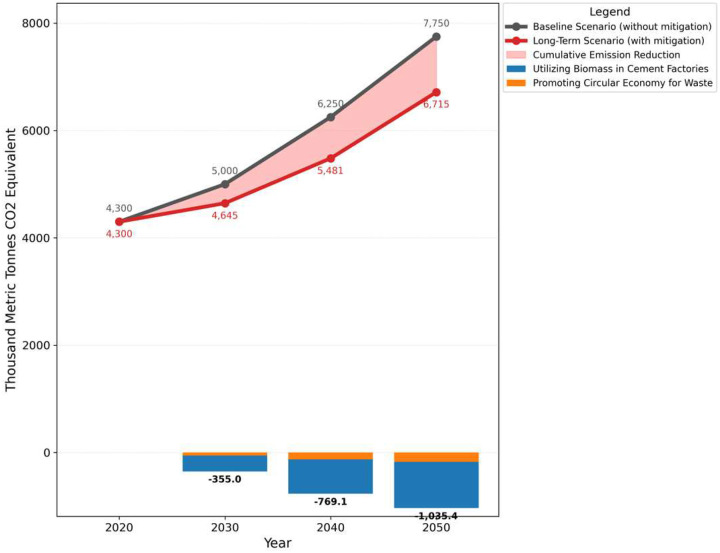
100-Year GWP: Direct (At Point of Emissions) Scenario: Long Term scenario, Effect: Methane

The long-term plan emphasized the use of alternative fuels and circular economy concepts in addition to sustainable waste management techniques. It is anticipated that emissions will have increased from the baseline of 6250.37 thousand metric tons in 2040 to 7769.88 thousand in 2050. Promoting a Circular Economy for Waste, including Source Separation of Waste, would be one of the major initiatives that decrease emissions by 3267 thousand metric tons in 2030 and 7753 thousand metric tons by 2050 as shown in figure 5. The largest reduction in emissions would be achieved by using biomass in cement factories which decreased emissions by 163.34 thousand metric tons in 2030 and 387.64 by 2050. These projects’ combined impact decreased emissions from 7769.88 to 7304.72 thousand metric tons by 2050, underscoring the significance of long-term sustainable waste management techniques.

**Fig 6:**
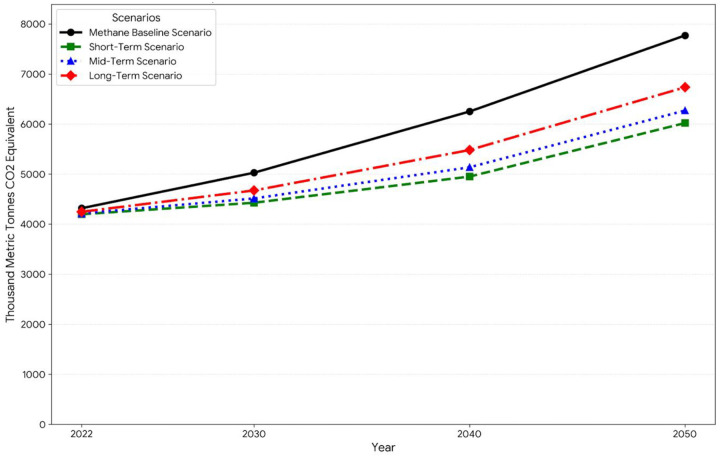
100-Year GWP: Direct (At Point of Emissions) Comparison of all Methane emission scenarios (2020–2050)

The LEAP models time-framed scenarios offer a thorough framework for assessing how well waste management initiatives lower methane emissions. The findings highlight the significance of a phased strategy that combines short-term initiatives with long-term plans to meet climate change mitigation and sustainable waste management objectives as shown in figure 6. This increasing trend highlights how urgently efficient waste management techniques are needed to reduce methane emissions, a powerful greenhouse gas that is causing climate change

### 3.3 Scenario-Based Analysis of Methane Emission Reduction Strategies

Methane emissions can be significantly reduced through targeted waste management practices according to a scenario-based analysis of strategies for reducing methane emissions for Jordan’s waste management system beginning in 2025.

Furthermore, Table 6 shows the relative efficacy of each strategy by offering a thorough numerical comparison of the yearly methane emissions for each scenario from 2025 to 2050.

The Baseline Scenario predicts a steady increase in annual methane emissions from 59500 tons in 2025 to 93500 tons by 2050, assuming no major changes in waste management practices.

The scenario that reduces methane emissions the most out of the four that were evaluated is the Incineration with Energy Recovery scenario, which is predicting emissions drop from 59500 tons in 2025 to 44900 tons by 2050. This indicates a decrease of roughly 52% compared to baseline.

Significant emission reductions are also shown in the Composting Focus scenario, where methane emissions drop to 49500 tons by 2050, a decrease of roughly 47% from the baseline. The anaerobic decomposition of organic matter, a significant source of methane emissions, can be reduced by diverting organic waste from landfills to composting facilities as this scenario emphasizes. But after 2040 the efficacy of these strategies plateaus, indicating that although composting is a useful tool, it might need to be combined with other tactics for long-term effects.

In the Anaerobic Digestion scenario, emissions are projected to drop to 53300 tons by 2050 which is a 43% decrease from the baseline.

Although it is successful in lowering emissions when compared to the baseline, the Recycling Focus scenario exhibits the least reduction of the four strategies, with emissions reaching 60400 tons by 2050—a reduction of 35%.

**Fig. 7:**
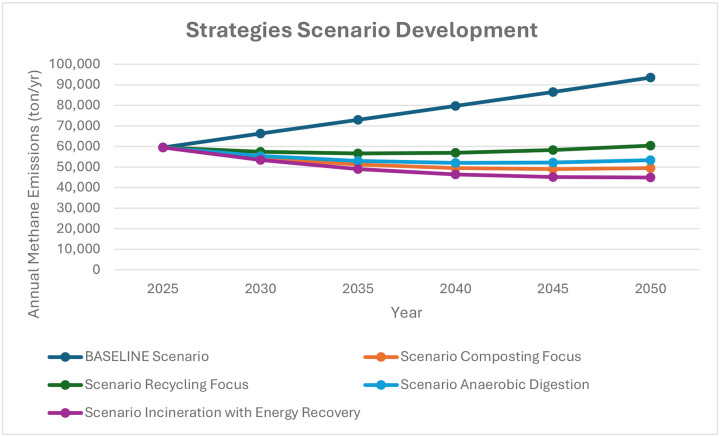
All scenarios for annual methane Emissions(ton/yr) in Jordan by using WasteMAP

The emission trends across all strategies are graphically summarized in Figure 7, which provides a thorough comparison of all scenarios.

### 3.3 Public Health and Environmental Impacts of Solid Waste Management

The findings show that pollution from current waste management practices has a substantial impact on public health, and therefore the economy, throughout the period from 2020 and 2050 (Kaza et al., 2018; Landrigan et al., 2017). Between 2020 and 2050 the economic damage associated with methane emissions, primarily coming from mortality costs estimated using the VSL, increases exponentially from 1.94 billion to 40.94 billion.

Based on the typical global warming potential (GWP) factors (which is assumed to be 25, common in the waste sector’s reporting) and the health effects of global warming according to the UNEP Global Methane Assessment (UNEP,2021, MoE,2019), table 7 summarizes the results. These figures represent the specific mortality and morbidity associated with the role of methane in producing ozone at the ground level (smog).

The data indicates a progressive deterioration in public health outcomes corresponding to the projected rise in landfill emissions. In the baseline scenario, the physical mass of methane emitted is expected to grow from approximately 173 thousand metric tons in 2022 to over 310 thousand metric tons by 2050. This 80% increase in emissions directly translates to a near-doubling of associated health burdens. Specifically, premature deaths attributed to ozone exposure from this specific source are projected to rise from 245 per year in 2022 to 440 per year in 2050. Similarly, respiratory morbidity, measured by asthma-related hospital visits, is expected to surge from roughly 744 annual cases to over 1,300 by the mid-century mark, highlighting a compounding burden on healthcare systems if the baseline scenario remains unchecked.

For someone willing to pay $300 to reduce risk of death by 1 in 10,000, for example, the VSL would be $3 million ($300 divided by 1 in 10,000). In countries like Jordan, where direct VSL studies are scarce, figures are usually changed from research done in higher-income nations, adjusted for economic inequalities. This is achieved via an income-elasticity component since people in higher-income countries typically demonstrate a greater WTP to reduce mortality risk. Here we are using a VSL of $7.2 million, used in most studies by United States government agencies, and then adjusting it using a 0.85 income-elasticity. This generates a VSL for application in Jordan of $1.16 million in 2019. Income in Jordan was adjusted using the Gross National Income (GNI)-per-capita modification to account for income produced locally and abroad. We note that meta-analyses estimate a worldwide average VSL of $1.3 million, which is in the range of the value estimated for Jordan. Therefore, a VSL of $1.3 million is adopted for Jordan in this study for the year 2022, we are using $1.3 million for consistency with this recent, Jordan-specific study (Rashdan, 2024).

## 4 Discussion

The dynamic character of waste generation and disposal is emphasized by these variations, underscoring the necessity of a thorough comprehension of the fundamental forces influencing these patterns. Considering methane’s significant greenhouse gas effects, these forecasts emphasize the urgent necessity for proactive waste management methods to reduce emissions and facilitate initiatives targeted at climate change mitigation. Jordan’s reliance on landfilling will continue to increase environmental and public health risks .

To address these problems, integrated waste management techniques that prioritize sustainability must be modified. Composting, expanding recycling and garbage-to-energy solutions can significantly reduce methane emissions and the quantity of waste dumped in landfills. To develop adaptable solutions that ensure long-term waste management, policy plans should also take demographic and economic factors into account. The results demonstrate the importance of pursuing a circular economy, which reduces waste, recovers resources and successfully manages emissions. So that it aligns with global and national sustainability goals.

A comprehensive strategy incorporating sustainable waste management techniques, smart emission monitoring technologies, and a health-centric approach is required to address Jordan’s municipal solid waste management issues. By aligning Jordan’s waste management strategies with global sustainability goals and utilizing advanced technologies such as LEAP and WasteMAP, the country can improve air quality, reduce the adverse effects of waste-related emissions, and slow down global warming (De La Barrera & Hooda, 2016).

The findings show that methane emissions from MSW in landfills can be considerably decreased by combining short- mid- and long-term waste management scenarios. Recycling and methane gas collection were examples of short-term scenarios, transfer stations and engineered sanitary landfills were examples of mid-term scenarios. Long-term scenarios provided viable ways to further reduce emissions, especially the use of organic waste in composting facilities and the encouragement of a circular economy.

The study does, however, also point out certain difficulties, including the need for stakeholder coordination, high upfront costs, and the need for technical expertise. The anticipated drops in methane emissions highlight how better waste management techniques may help mitigate climate change despite these obstacles. To optimize these projects and overcome implementation obstacles, future research should concentrate on this area.

Methane is a climate pollutant with an atmospheric lifetime of 12 years, meaning it is considered short-lived. In Jordan, methane emissions from municipal solid waste, especially landfills, were 4,317.58 thousand metric tons in 2020 and are expected to increase to 7,769.88 thousand metric tons of carbon dioxide equivalent by 2025, and it is a major precursor of tropospheric ozone through its photochemical reactions with hydroxyl radicals (OH) in the presence of nitrogen oxides (NOₓ).

We consider alternative strategies for disposing of solid waste that reduce methane emissions and can also provide other environmental advantages. For example anaerobic digestion and composting mitigate methane emissions while simultaneously producing biogas and compost byproducts (Abu Qdais et al, 2019).

Long-term exposure to high levels of ozone increases the risk of chronic obstructive pulmonary disease (COPD) and premature death. Tropospheric ozone is a harmful air pollutant and causes several diseases, including cardiovascular and respiratory diseases and mortality. The World Health Organization (WHO) recommends that ozone concentrations not exceed 60 μg/m^3^ (30 parts per billion) during the peak season (WHO, 2021).

This increase demonstrates the rapidly mounting public health burden (see also Fuller et al., 2022) from methane and shows that many of the improved waste management strategies we study here are likely to be highly cost-effective in addition to their environmental merit. The financial burden from the health consequences of methane emission will continue to rise if no changes are made in solid waste management.

These health effects are primarily caused by the chemical function of methane as a precursor to ground-level ozone, rather than the direct toxicity of the gas itself inhaled at the landfill site. When methane is released into the atmosphere, it combines with sunlight and other pollutants to produce ozone. This chemical is a powerful respiratory irritant that causes inflammation of the lungs, decreased lung capacity, and aggravation of chronic diseases like asthma and emphysema. The original data was provided in “CO2 equivalent”, a metric designed to account for climate change. However, converting this data to mass reveals that the volume of methane that is directly released into the atmosphere is significant enough to contribute to the degradation of regional air quality. As a result, reducing the emission of landfills has a co-benefit: it not only decreases the global warming potential associated with the original table, but also has immediate, demonstrable health benefits via the lowering of mortality and hospitalizations associated with ozone.

Reducing global anthropogenic methane emissions by 45% by 2022 would prevent approximately 255,000 premature deaths annually due to reduced ozone exposure, according to the UNEP and CPCC Global Methane Assessment (2021). According to the study, the health benefit was estimated at 1,400 deaths avoided per million tons of methane reduced (UNEP and CCAC, 2021).

Reducing methane emissions in the waste sector should be considered a public health measure and a firm priority in climate policy. Alternative waste management, recycling, and methane capture will also directly contribute to reducing ozone levels, thus aligning Jordan’s activities with international and national sustainability goals.

The public health benefits are not only qualitative but also measurable in economic terms. If no interventions are applied, the costs in human life in Jordan—as quantified by the Value of Statistical Life (VSL)—represent significant losses due to premature deaths and the disease burden linked to poor waste management. Each intervention scenario demonstrates potential to save lives and reduce healthcare expenditures. Short-term actions lower immediate health risks, mid-term improvements provide systemic protection for urban and rural populations, and long-term strategies generate the greatest savings in the greatest savings in avoided VSL costs, representing an estimated $22.85 billion in avoided annual mortality costs by 2050. These results emphasize that effective waste management is not only an environmental necessity but also a public health imperative that safeguards lives, reduces medical costs, and ensures alignment with both national and global sustainability goals.

The research reveals that methane emissions from municipal solid waste in Jordan provide both an environmental concern and a substantial public health and economic cost. Under the baseline scenario, death costs associated with methane emissions are anticipated to escalate significantly from 1.94 billion USD in 2020 to 40.94 billion USD by 2050. This significant rise underscores the severity of the issue if no measures are implemented, with expenses increasing concurrently with population and economic expansion.

The current research measures mortality-related damage using the Value of a Statistical Life (VSL). However, these values reflect a conservative estimate of the actual economic impact of landfill emissions. Supplementary expenses, including healthcare costs associated with morbidity, reduced labour productivity, and the depreciation of property values next to dump sites, are not currently included in these assessments. Furthermore, the long-term forecasts extending to 2050 must be approached with care. Jordan’s per capita income is anticipated to increase significantly in the next decades, which, due to the income elasticity of VSL, would greatly increase the monetized harms linked to premature death. The anticipated 40.94 billion USD cost by 2050 is therefore conservative; real future costs may be much larger.

Situating these conclusions within a wider international framework accentuates their policy significance. Evidence from nations like China and the United States indicates that with rising population and affluence, the public’s demand for landfill emission regulations and expenditures in waste management infrastructure will escalate. These comparisons demonstrate how Jordan may encounter increasing social and political challenges if income levels increase. Divergent socio-economic pathways remain feasible simultaneously: the most adverse results would arise in circumstances of fast population increase coupled with stagnating income, resulting in elevated waste creation, and heightened sensitivity to pollution. Subsequent studies should assess a range of pathways—encompassing income, population, and policy variables—to provide a more thorough understanding of the possible health and economic repercussions of landfill emissions.

The substantial diversion of non-recyclable waste to energy recovery facilities, which not only lowers the amount of waste dumped in landfills but also produces energy, is responsible for the strategy’s success. This helps to reduce emissions and promote energy sustainability. This scenario is a promising choice for nations like Jordan, where energy recovery from waste can support energy security as it capitalizes on the dual advantages of biogas production and methane emission reduction. Anaerobic digestion might not be enough to meet the most aggressive emission reduction goals when compared to incineration. Although recycling is crucial in lowering the amount of waste that needs to be disposed of, this scenario shows that recycling may not be enough to solve the methane problem on its own especially when it comes to organic waste, given its limited effect on methane emissions.

The results indicate that the most effective strategy to reduce methane emissions from waste management in Jordan may involve a combination of strategies, particularly those that prioritize composting and incineration with energy recovery. Although each scenario has an advantage, the best results are probably obtained by combining several strategies that are specific to the waste composition and local conditions. Future studies could look into the social and economic feasibility of these strategies as well as any possible synergies to provide a more comprehensive framework for sustainable waste management in Jordan and similar contexts.

Overall, the results show public health is affected by the economic and environmental effects of poor waste management and how they are interconnected (Kaza *et al*., 2018). Escalating economic losses and rising temperatures (Calvin *et al*., 2023) underscore the pressing need for comprehensive action during public awareness campaigns for technological advancements and policy interventions are crucial to reducing these effects (Da Silva *et al*., 2020). If nothing changes, the expenses will keep going up, and the planet and human health will suffer irreparable harm. Stricter emissions regulations, investments in greener technologies and the promotion of sustainable waste management techniques (Kirchherr, Reike and Hekkert, 2017) are just a few of the many strategies needed to address these problems.

## 5 Conclusions

### 5.1 Conclusion

Public health air quality and climate change are all significantly impacted by municipal solid waste management (MSWM), which is a crucial issue in Jordan. The study emphasizes how efficient waste management techniques are urgently needed to reduce methane emissions of a powerful greenhouse gas, and enhance public health. The study assessed several waste management techniques, such as anaerobic digestion, composting, recycling, and incineration with energy recovery, using scenario-based analysis and the LEAP model. The results show that the most efficient way to lower methane emissions is to combine tactics, especially composting and incineration with energy recovery. The time-framed scenarios also show that methane emissions from landfills can be considerably decreased by using a phased approach that incorporates short- mid- and long-term scenarios. Notwithstanding obstacles like high upfront costs and the need for technical know-how, the anticipated savings highlight how better waste management techniques can help mitigate climate change and enhance public health.

Ultimately, the results of this study have an international scope. The scenarios that are analyzed here suggest that Jordan is a viable study case for countries that are experiencing semi-arid weather conditions and have to deal with the dual effects of urbanization and refugees. The discovery that combined strategies that involve circular economy principles and energy recovery have the greatest health and economic benefits suggests a valid path for similar economies that want to align their waste with global goals regarding climate.

### 5.2 Recommendations

Take a Multi-Strategy Approach: To optimize methane emission reductions, Jordan should employ a mix of waste management techniques such as anaerobic digestion composting and incineration with energy recovery. Local conditions and waste composition should be considered when designing this integrated approach. Prioritize Short-Term scenario: To minimize methane emissions quickly, measures like the Recycling Project for Organic Waste Processing Plant and Methane Gas Collection at Al-Ekaider Landfill should be given top priority. Invest in Mid-Term scenario: To build on the successes of short-term scenario and guarantee long-term emission reductions, mid-term scenario such as converting landfills into engineered sanitary landfills and creating waste transfer stations, should be put into action. Encourage Long-Term Sustainable Practices: To ensure sustainable waste management and further reduce methane emissions, long-term strategies such as adoption of a circular economy and using organic waste in composting facilities should be pursued. Improvement of Stakeholder Coordination: The successful execution of waste management projects depends on efficient coordination between stakeholders, including local communities, businesses, and government organizations. To aid in these endeavors, laws and policies ought to be reinforced. Leverage Global Best Practices: Jordan should take advantage of global sustainability standards and best practices by tracking and lowering methane emissions with the help of programs like Waste Management and Prevention Lifecycle Assessment (WasteMAP) and the Low Emission Analysis Platform (LEAP). Research in the Future: To optimize waste management initiatives and overcome implementation obstacles, future studies should concentrate on this area. To get more support, studies should also investigate the socioeconomic advantages of these initiatives.

## Supplementary Files

This is a list of supplementary files associated with this preprint. Click to download.

- DataSustainabilityinJordansMunicipalSolidWasteManagement1.docx

## Figures and Tables

**Table 1:** Municipal Solid Waste Data for Jordan.

Items	Quantity for 2019 (thousand tons/a)	Quantity for 2020 (thousand tons/a)	Quantity for 2021 (thousand tons/a)	Quantity for 2022 (thousand tons/a)	Quantity for 2023 (thousand tons/a)
Waste Landfilled according to MoLA	1,663	1,795.4	2,649.5	2,914.4	1,732.4
Waste buried in landfill boundaries of the GAM	1,456	1,374.8	1,350.5	1,368.9	1,406
Total (MSW) Disposed in Landfills in Jordan	3119	3,251.4	4,105.5	4370.5	3188.5

**Table 2:** Population Growth Data with Disease Date and economic data for Jordan.

Data	Units	2022	2030	2040	2050
Population	m People	11.345	13.45124	16.72135	20.78645
Population Growth	%/ annum	2	2.32909	2.895311	3.599185
Respiratory Disease Ozone Relative Risk	Rate (%)	1.04	1.04	1.04	1.04
GDP	USD	49.375	60.210	76.325	96.754
GDP Growth	%/a	2.4	2.83342	3.591786	4.55313
Average Income	USD/Person	4352.1375	4476.179	4564.55	4654.667

**Table 3:** Summary of listing of different mitigation options of methane reduction in Jordan (2020 to 2050).

Action Number	Action Title
Short-Term Scenario	
1	Biogas from Salt Treatment Plant
2	Recycling Bank Project in Amman
3	Organic Waste Processing Plant (MBT) in Amman
4	Regulation of Informal Waste Collectors
5	Enhancing Operations of RUA Waste Management Company
6	Methane Gas Collection at Al-Ekeider Landfill
7	Management of Studies and Awareness on Solid Waste
Sub-Total short-term actions	
Mid-Term Scenario	
1	Transforming Landfills into Engineered Sanitary Landfills
2	Development of Waste Transfer Stations
3	GPS-Based Guidance System
4	National Waste Management Center
5	Biogas Collection from Al-Husainiyat Landfill
Sub-Total Mid-term actions	
Long-Term Scenario	
1	Promoting Circular Economy for Waste
2	Utilizing Biomass in Cement Factories

**Table 4:** Inventory of Methane Baseline Scenario.

Branch: Non-Energy\Waste\Methane from MSW in Landfills Units: Thousand Metric Tons CO_2_ Equivalent				
Year	2022	2030	2040	2050
Methane Base line Scenario	4,317.58	5,028.02	6,250.37	7,769.88

**Table 5:** Summary of the activities’ impact on methane reduction in Jordan (2020 to 2050).

Action Number	Action Title	Methane Reduction (Gg CO_2_ in 2050)
	*Short-Term Scenario*	
*1*	Biogas from Salt Treatment Plant	19.87
*2*	Recycling Bank Project in Amman	301.68
*3*	Organic Waste Processing Plant (MBT) in Amman	301.68
*4*	Regulation of Informal Waste Collectors	150.84
*5*	Enhancing Operations of RUA Waste Management Company	150.84
*6*	Methane Gas Collection at Al-Ekeider Landfill	754.2
*7*	Management of Studies and Awareness on Solid Waste	150.84
	*Sub-Total short-term actions*	**1810.08**
	*Mid-Term Scenario*	
*1*	Transforming Landfills into Engineered Sanitary Landfills	876.44
*2*	Development of Waste Transfer Stations	175.29
*3*	GPS-Based Guidance System	87.64
*4*	National Waste Management Center	175.29
*5*	Biogas Collection from Al-Husainiyat Landfill	175.29
	*Sub-Total Mid-term actions*	**1,489.94**
	*Long-Term Scenario*	
*1*	Promoting Circular Economy for Waste	172.56
*2*	Utilizing Biomass in Cement Factories	862.81
	*Sub-Total Long-term actions*	**1035.37**
*Total Reduction in Waste and Wastewater Sector*		**4335.39**

**Table 6:** Yearly Methane Emissions Strategies Scenarios (2025–2050)

unit is Annual Methane Emissions (ton/yr)	Year					
	2025	2030	2035	2040	2045	2050
*BASELINE Scenario*	59,500	66,300	73000	79700	86500	93500
*Scenario Composting Focus*	59,500	54500	51200	49500	49000	49500
*Scenario Recycling Focus*	59,500	57400	56600	56900	58300	60400
*Scenario Anaerobic Digestion*	59,500	55400	53000	52000	52200	53300
*Scenario Incineration with Energy Recovery*	59,500	53400	49000	46400	45100	44900

**Table 7:** Estimated Health Impacts of Landfill Methane Emissions

YEAR	METHANE MASS (CH4) (THOUSAND METRIC TONS)	EST. PREMATURE DEATHS(ANNUAL)	EST. ASTHMA HOSPITAL VISITS(ANNUAL)
**2022**	173	245	744
**2030**	201	285	866
**2040**	250	354	1,076
**2050**	311	440	1,338

## Data Availability

The datasets generated during and/or analysed during the current study are available in the Zenodo repository, Ghawanmeh, A. (2026). Sustainability in Jordan’s Municipal Solid Waste Management. Zenodo. https://doi.org/10.5281/zenodo.18624544
